# Mesenchymal stromal cell-derived nanovesicles ameliorate bacterial outer membrane vesicle-induced sepsis via IL-10

**DOI:** 10.1186/s13287-019-1352-4

**Published:** 2019-08-01

**Authors:** Kyong-Su Park, Kristina Svennerholm, Ganesh V. Shelke, Elga Bandeira, Cecilia Lässer, Su Chul Jang, Rakesh Chandode, Inta Gribonika, Jan Lötvall

**Affiliations:** 1 Krefting Research Centre, Institute of Medicine, University of Gothenburg, 40530 Gothenburg, Sweden; 2 Department of Anesthesiology and Intensive Care Medicine, Institute of Clinical Science, Sahlgrenska Academy, University of Gothenburg, 40530 Gothenburg, Sweden; 3 Codiak BioSciences Inc, 500 Technology Square, 9th floor, Cambridge, MA 02139 USA; 4 Department of Microbiology and Immunology, Institute of Biomedicine, University of Gothenburg, 40530 Gothenburg, Sweden

**Keywords:** Extracellular vesicles, Nanovesicles, Mesenchymal stromal cells, Outer membrane vesicles, Sepsis, Anti-inflammation

## Abstract

**Background:**

Sepsis remains a source of high mortality in hospitalized patients despite proper antibiotic approaches. Encouragingly, mesenchymal stromal cells (MSCs) and their produced extracellular vesicles (EVs) have been shown to elicit anti-inflammatory effects in multiple inflammatory conditions including sepsis. However, EVs are generally released from mammalian cells in relatively low amounts, and high-yield isolation of EVs is still challenging due to a complicated procedure. To get over these limitations, vesicles very similar to EVs can be produced by serial extrusions of cells, after which they are called nanovesicles (NVs). We hypothesized that MSC-derived NVs can attenuate the cytokine storm induced by bacterial outer membrane vesicles (OMVs) in mice, and we aimed to elucidate the mechanism involved.

**Methods:**

NVs were produced from MSCs by the breakdown of cells through serial extrusions and were subsequently floated in a density gradient. Morphology and the number of NVs were analyzed by transmission electron microscopy and nanoparticle tracking analysis. Mice were intraperitoneally injected with *Escherichia coli*-derived OMVs to establish sepsis, and then injected with 2 × 10^9^ NVs. Innate inflammation was assessed in peritoneal fluid and blood through investigation of infiltration of cells and cytokine production. The biodistribution of NVs labeled with Cy7 dye was analyzed using near-infrared imaging.

**Results:**

Electron microscopy showed that NVs have a nanometer-size spherical shape and harbor classical EV marker proteins. In mice, NVs inhibited eye exudates and hypothermia, signs of a systemic cytokine storm, induced by intraperitoneal injection of OMVs. Moreover, NVs significantly suppressed cytokine release into the systemic circulation, as well as neutrophil and monocyte infiltration in the peritoneum. The protective effect of NVs was significantly reduced by prior treatment with anti-interleukin (IL)-10 monoclonal antibody. In biodistribution study, NVs spread to the whole mouse body and localized in the lung, liver, and kidney at 6 h.

**Conclusions:**

Taken together, these data indicate that MSC-derived NVs have beneficial effects in a mouse model of sepsis by upregulating the IL-10 production, suggesting that artificial NVs may be novel EV-mimetics clinically applicable to septic patients.

**Electronic supplementary material:**

The online version of this article (10.1186/s13287-019-1352-4) contains supplementary material, which is available to authorized users.

## Background

Advanced infectious diseases such as sepsis are associated with excessive release of cytokines into the systemic circulation, induced by an aggressive host immune response to an infection [1]. The incidence of sepsis has increased worldwide and is a major cause of death among all age groups [1, 2]. Therefore, the development of novel therapeutic approaches is crucial to improve clinical outcomes. A significant number of studies have reported that mesenchymal stromal cells (MSCs) could be an effective therapeutic candidate by promoting immunomodulation in the field of sepsis [3–7]. Treatment of patients with sepsis with MSCs of various origins has resulted in reduced mortality and improved myocardial function in a mouse sepsis model [6, 7]. This protective effect of MSCs has been mainly ascribed to the interaction of MSCs with immune cells such as macrophages in biological fluids and tissues, resulting in a diminished secretion of pro-inflammatory cytokines [6, 7].

Extracellular vesicles (EVs) are spherical bilayered proteolipid structures ranging in diameter from approximately 30 to 3000 nm and carry a specific subset of proteins, lipids, and genetic materials such as mRNAs, miRNAs, and DNAs [8–10]. EVs are recognized as mediators of advanced intercellular communication, as they are able to shuttle these multiple molecules between cells. Importantly, MSC-derived EVs to a large extent mimic the regenerative and immunosuppressive effect of the cells they are produced from, as shown in various disease models, including acute lung injury, acute kidney injury, and ischemia [11–13]. Especially, recent evidence have shown that MSC-derived EVs may be an attractive candidate for immunosuppressive therapy in sepsis [14, 15]. EVs have been shown to be protective against sepsis-associated cardiac injury mediated by vesicular miR-223 [14] and can prevent lethality and organ dysfunction induced by experimental cecal ligation puncture-induced sepsis [15].

Despite the therapeutic advantage of EVs, there are some limitations to be overcome for the application of EVs to the clinic. Most mammalian cells, including MSCs, secrete relatively low numbers of EVs per cell, and the totality of these natural EVs harbor a complex heterogeneity. These issues, and the time-consuming procedures to isolate EVs, may reduce the risk of low yield [16, 17]. Recent studies have shown that EV-mimetic nanovesicles (NVs) can be artificially developed from most cells with significantly higher production yield using serial extrusion through filters. Artificially produced NVs from monocytes/macrophages could be loaded with a chemotherapeutic drug or c-myc siRNA, and they could be targeted to malignant tumor leading to tumor regression [18, 19]. In addition, therapeutic insulin-producing cells could be well differentiated from initial bone marrow cells by NVs generated from pancreatic β cells [20]. Interestingly, NVs have also been demonstrated to be made from adipose-derived stem cells, and have regenerative effects in an emphysema model through FGF2 delivery [21]. However, whether MSC-derived NVs contribute to protection against sepsis remains to be clarified.

Many severe infections, both bacterial and viral, can induce a strong inflammatory cascade of cytokine release, often called “cytokine storm.” Gram-negative bacteria can induce such a host reaction by releasing outer membrane vesicles (OMVs) [22, 23], which distribute many virulent factors systemically, including lipopolysaccharides (LPS), lipoproteins, and genetic materials. OMVs can elicit pathophysiological function in bacteria-host interaction by modulating the host immune response [24, 25]. We have previously shown that OMVs provoke a sepsis-like inflammatory response and activate cardiomyocytes resulting in sepsis-associated cardiac dysfunction [26, 27]. We here aimed to elucidate whether MSC-derived NVs could have any therapeutic inhibitory effect in OMV-induced cytokine storm in the mouse. To study this, we generated NVs from human bone marrow-derived MSCs by subjecting cells to serial extrusion through micro-sized filters and characterized their morphology and contents by transmission electron microscopy (TEM) and mass spectrometry. Further, NVs were applied to OMV-exposed macrophages in vitro, as well as in mice in vivo, evaluating NV-mediated inhibitory effects on cytokine release.

## Materials and methods

### Animals

Wild-type mice of the C57BL/6 genetic background (6 weeks old) were purchased from Charles River. The mice were maintained at Experimental Biomedicine (EBM) at the University of Gothenburg, Sweden. The study was approved by the local Animal Ethics Committee in Gothenburg, Sweden (permit no 89-2016, 131-2016, 22-2016) and performed according to institutional animal use and care guidelines.

### Cell cultures

Human bone marrow-derived MSCs were purchased from ATCC (Manassas, VA) and maintained in Minimum Essential Media alpha GlutaMAX™ (Thermo Fisher Scientific, Waltham, MA) containing 10% fetal bovine serum (FBS), 100 U/mL penicillin, and 100 μg/mL streptomycin. MSCs were grown to 60–70% confluence and cultured at clonal density. RAW 264.7 cells were grown in Dulbecco’s modified Eagle’s medium (HyClone, Logan, UT) supplemented with 10% FBS, 100 U/mL penicillin, and 100 μg/mL streptomycin. U937 cells were grown in RPMI 1640 medium (Invitrogen, Carlsbad, CA) supplemented with 10% FBS, 100 U/mL penicillin, and 100 μg/mL streptomycin. Mouse splenic T cells and B cells were purified using cell isolation kit (Miltenyi Biotec, Bergisch Gladbach, Germany) according to the manufacturer’s instructions, and then grown in RPMI 1640 medium supplemented with 10% FBS, 25 mM HEPES, 1 mM sodium pyruvate, 50 μM beta mercaptoethanol, 100 U/mL penicillin, and 100 μg/mL streptomycin. All cells were cultured at 37 °C in an atmosphere of 5% CO_2_.

### Preparation of NVs

MSC-derived NVs were prepared using the previous protocol with some modification [18, 20]. Low passage MSCs (passage five) were detached from plates when they reached 60–70% confluence. The cell suspension in phosphate-buffered saline (PBS) was sequentially extruded five times through 10-, 5-, and 1-μm pore-sized polycarbonate membrane filters (Whatman, Dassel, Germany) using an extruder (Avanti Polar Lipids, Birmingham, AL). Respectively 1 and 2 mL of 50 and 10% solution of iodixanol (Axis-Shield PoC AS, Oslo, Norway), followed by 7 mL of the cell suspension from the membrane filter, were sequentially added to each 10-mL ultracentrifuge tube. The layers formed between 50% iodixanol and 10% iodixanol after ultracentrifugation at 100,000×*g* for 2 h at 4 °C was collected and considered as NVs. Total of three batches of NVs were isolated and tested the therapeutic activities on macrophages (Additional file 1: Figure S1A, S1B). Based on that they have similar potency to each other, the same batch of NVs was used in the following experiments.

### Preparation of OMVs derived from *Escherichia coli*

A uropathogenic *E. coli* strain was used to produce and isolate OMVs. *E. coli* OMVs were isolated using the protocol described previously with modification [28]. Bacterial suspensions were pelleted at 6,000×*g*, 4 °C for 20 min, twice, and then the supernatant was filtered through a 0.45-μm vacuum filter and was concentrated by ultrafiltration Vivaflow 200 module (Sartorius, Goettingen, Germany) with a 100-kDa cut-off membrane. The remaining solution was subjected to ultracentrifugation at 150,000×*g*, 4 °C for 3 h and resuspended with PBS.

### TEM

NVs were investigated by negative stain electron microscopy. Concretely, NVs were blotted for 5 min onto glow-discharged 200-mesh, formvar carbon-coated copper grids (Electron Microscopy Sciences, Hatfield, PA). Then, NVs were washed with water, followed by fixed using 2.5% glutaraldehyde dissolved PBS. After further washing with water, the samples were stained using 2% uranyl acetate for 1.5 min. Negative-stained NVs were observed on a digitized LEO 912AB Omega electron microscope (Carl Zeiss SMT, Oberkochen, Germany) at 120 kV with a Veleta CCD camera (Olympus-SiS, Stuttgart, Germany).

### Nanoparticle tracking analysis

NVs (10 μg/mL) were dispersed in PBS, and then the particle concentration of NVs was assessed by ZetaView analyzer (Particle Metrix GmbH, Meerbuch, Germany). Measurements were evaluated in triplicates, and each individual data was acquired from two stationary layers with five times measurements in each layer. Sensitivity of the camera was configured at 70 in all measurements. Data were analyzed using ZetaView analysis software version 8.2.30.1.

### LC-MS/MS analysis

Three biological replicate NVs (30 μg) were digested with trypsin using the filter-aided sample preparation (FASP) method [29] and C18 spin columns desalting according to the manufacturer’s instructions. All fractions were dried on Speedvac and reconstituted in 3% acetonitrile and 0.2% formic acid and analyzed on an Orbitrap Fusion Tribrid mass spectrometer interfaced with Easy-nLC 1200 (Thermo Fisher Scientific, Waltham, MA). Peptides were trapped on the Acclaim Pepmap 100 C18 trap column (100 μm × 2 cm, particle size 5 μm; Thermo Fischer Scientific) and separated on the in-house packed C18 analytical column (75 μm × 30 cm, particle size 3 μm) using the gradient from 5 to 32% B in 75 min and from 32 to 100% B in 5 min; solvent A was 0.2% formic acid and solvent B was 80% acetonitrile and 0.2% formic acid. Precursor ion mass spectra were recorded at 120 000 resolution, the most intense precursor ions were selected, fragmented using collision-induced dissociation (CID) at collision energy setting of 35; spectra and the MS/MS spectra were recorded in ion trap with the maximum injection time of 40 ms and the isolation window of 0.7 Da. Charge states 2 to 7 were selected for fragmentation; dynamic exclusion was set to 45 s with 10 ppm tolerance. MS3 spectra for reporter ion quantitation were recorded at 50 000 resolution with HCD fragmentation at collision energy of 60 using the synchronous precursor selection of the 7 most abundant MS/MS fragments, with the maximum injection time of 100 ms.

### Database search

Data analysis was performed using Proteome Discoverer version 2.2 (Thermo Fisher Scientific, Waltham, MA). The database search was performed against the Swissprot *Homo sapiens* database. Mascot 2.5.1 (Matrix Science, London, UK) was used as a search engine with precursor mass tolerance of 10 ppm and fragment mass tolerance of 0.6 Da; one missed cleavage was accepted, mono-oxidation on methionine was set as a variable modification, and methylthiolation on cysteine was set as a fixed modification. Percolator was used for the validation of identification results with the strict target false discovery rate of 1%, and proteins were only considered when identified in all three replicates. For protein class analysis and pathway classification, Panther classification system was used (http://pantherdb.org/). Gene ontology (GO) analysis was obtained using DAVID (https://david.ncifcrf.gov/) and Funrich analysis tool.

### Western blot analysis

NVs (10 μg) and whole-cell lysates (25 μg) were separated by 10% SDS-PAGE and transferred to a polyvinylidene difluoride membrane. The membrane blocked in nonfat milk was incubated with anti-flotillin-1 antibody (BD Biosciences, San Jose, CA), anti-CD81 antibody (Santa Cruz Biotechnology, Santa Cruz, CA), or anti-beta-actin antibody (Sigma Aldrich, St. Louis, MO). The immunoreactive bands were analyzed with a chemiluminescent substrate following incubation with horseradish peroxidase-conjugated secondary antibody.

### Uptake of NVs

NVs were stained with DiO (Molecular Probes, Eugene, OR) for 20 min at 37 °C. Cellmask Deep Red (Thermo Fisher Scientific, Waltham, MA)-labeled RAW 264.7 cells were incubated with DiO-labeled NVs for 6 h. The cells were fixed with 4% paraformaldehyde, and then permeabilized with 0.2% Triton X-100, followed by mounted with Prolong Gold antifade reagent (Thermo Fisher Scientific, Waltham, MA). The uptake was observed by a fluorescence light microscope (Zeiss Axio observer; Carl Zeiss, Oberkochen, Germany). For the uptake inhibitor treatment, RAW 264.7 cells pretreated with dynasore (Sigma Aldrich, St. Louis, MO) for 1 h were sequentially incubated with DiO-NVs for 6 h. Flow cytometry was analyzed using BD FACSVerse Flow Cytometer running BD FACSuit Software (BD Biosciences, San Jose, CA) and FlowJo Software (Tree Star Inc., Ashland, OR).

### In vivo assessment

Same batch of NVs was used in mice, and the following in vivo experiments were repeated twice. Mice were intraperitoneally (i.p.) injected with OMVs (15 μg in PBS, 100 μL) to provoke sepsis as previously described [30], and then administrated with NVs (2 × 10^9^) intraperitoneally. Mice were sacrificed at 6 h following anesthetization with i.p injection of xylazine chloride (10 mg/kg; Bayer, Gothenburg, Sweden) and ketamine hydrochloride (100 mg/kg; Pfizer AB, Kent, UK). Rectal temperature was measured by a thermometer (Bioseb, Chaville, France). Peritoneal fluid, blood, and bronchoalveolar lavage (BAL) fluid were collected from mice, and then the supernatants were stored at − 80 °C for cytokine analysis following centrifugation. The pelleted cells were analyzed using flow cytometry and light microscopy. For the analysis of surface marker expression, freshly isolated cells from the peritoneum were stained with fluorochrome-conjugated antibodies. Viable cells were blocked for non-specific staining with 2.4G2 (anti-Fc-receptor) and stained with CD25-PerCP-Cy5.5, CD4-BV786, GITR-PeCy7, MHCII-AF700, CD45-APC-Cy7, TGF beta-PE, Ly6G BV650, and Ly6C BV605 (BD Biosciences, San Jose, CA). Then, the Cytofix/Cytoperm kit (PharMingen, San Diego, CA) was used for intracellular IL-10 staining with IL-10-APC according to the manufacturer’s instructions. To exclude dead cells, 7-aminoactinomycin D (Sigma Aldrich, St. Louis, MO)-stained positive cells were excluded from the analysis. Events were collected and analyzed by using an LSR-II Flow cytometer (BD Biosciences, San Jose, CA) and FlowJo software (Tree Star Inc., Ashland, OR). For leukocytes and platelets counting in blood, blood samples were obtained by cardiac puncture, followed by putting into EDTA tubes. The number of leukocyte and platelets were counted using the light microscopy following incubation with 1% hydrochloride and Rees-Ecker diluting fluid (Thermo Fisher Scientific, Waltham, MA), respectively. The concentrations of cytokines and chemokines in serum were measured using the DuoSet ELISA Development kit and Proteome profiler cytokine array kit (R&D Systems, Minneapolis, MN). Neutralizing antibody experiments used rat IgM anti-mouse IL-10 mAb (200 μg per body; U-CyTech Biosciences, Utrecht, the Netherlands) or an isotype control antibody (Thermo Fisher Scientific, Waltham, MA).

### In vivo imaging of NVs

Firstly, NVs were incubated with 5 μM of Cy7 mono NHS ester (Amersham Biosciences, Little Chalfont Bucks, UK) for 30 min at 37 °C. Cy7-labeled NVs (2 × 10^9^) were injected i.p. to mice, and then Cy7 fluorescence in the mice was obtained by IVIS spectrum (Perkin-Elmer, Waltham, MA). Mice were sacrificed after 6 h and 24 h injection, and the fluorescence was acquired in various tissues, followed by measuring of radiant efficiency with Living Image 3.1 software.

### Cytokine release by macrophages in vitro

RAW 264.7 cells were seeded in a 24-well plate, and then OMVs (100 ng/mL) were treated for 3 h to induce inflammation. Various doses of NVs were added to the cells, and the supernatant concentrations of TNF-α, IL-6, and IL-10 at 15 h later were measured by DuoSet ELISA Development kit (R&D Systems, Minneapolis, MN). In the case of U937, the cells were differentiated into macrophages by incubation with 10 ng/mL PMA (Calbiochem, San Diego, CA) for 24 h. The macrophages were seeded in 24-well plate, and then OMVs (100 ng/mL) were treated for 3 h. NVs (1 × 10^9^) were added to the cells, and the supernatant concentrations of cytokines at 15 h later were measured. For peritoneal macrophages, 3 mL of sterile PBS was injected to the abdomen of each mouse, and then 2 mL peritoneal lavage was taken. The PBS wash step was repeated two more times, and the resulting lavage solution was centrifuged at 300×*g*, 4 °C for 5 min. The pellets were resuspended in RPMI 1640 medium supplemented with 10% FBS, 100 U/mL penicillin, 100 μg/mL streptomycin, and 50 μM beta mercaptoethanol, and seeded in 24-well plate. OMVs (100 ng/mL) were treated for 3 h, followed by the addition of NVs (1 × 10^9^) for 15 h.

### Statistical analysis

Values of results were tested for normal distribution (Shapiro-Wilk test or D’Agostino-Pearson normality test) and were expressed as the mean and standard error of the mean (SEM). One-way ANOVA followed by Tukey’s multiple comparison test was used to assess the difference between groups. The nonparametric Kruskal-Wallis test followed by Dunn’s multiple comparison was used when data did not pass the normality test. *P* < 0.05 was considered to be significant.

## Results

### Production and characterization of MSC-derived NVs

**Fig. 1:**
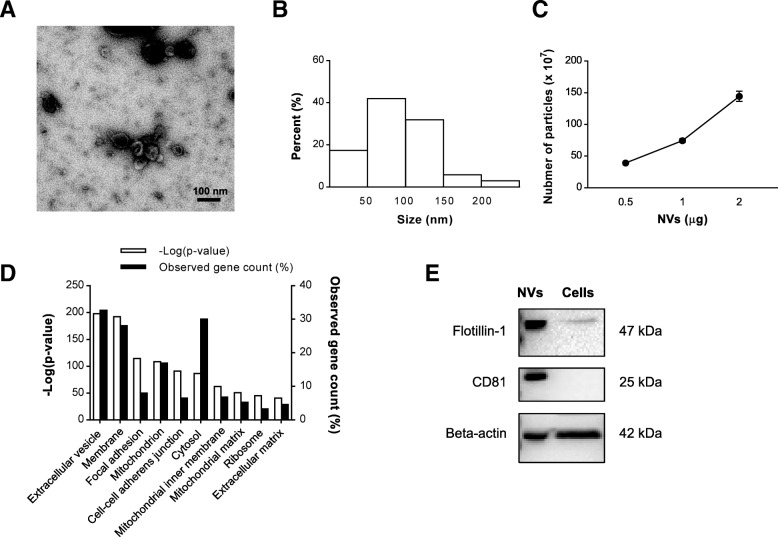
Characterization of MSC-derived NVs. **a** TEM image of purified NVs. Scale bar, 100 nm. **b** Size distribution of NVs according to diameter. **c** The number of particles per proteins of NVs measured by nanoparticle tracking analysis (*n* = 3). **d** Top 10 subcellular localization GO terms enriched in NV proteome (based on *P* value). Three biological replicates were used, and commonly identified proteins in three runs were analyzed for GO terms. **e** Western blot analysis of EV markers on NV (10 μg) and MSC lysates (25 μg)

NVs were isolated from MSCs according to the procedure described in the “Materials and methods” section. Briefly, early passages of MSCs were gathered and serially extruded through a variety of pore-sized polycarbonate membrane filters (10, 5, and 1 μm) to produce nano-sized vesicles. These NVs, isolated from the interface layers between the 10% and 50% iodixanol following two-step OptiPrep density gradient ultracentrifugation, were visualized by TEM showing spherical structures (Fig. 1a). The majority of the produced NVs have a diameter of 50–150 nm (Fig. 1b), and nanoparticle tracking analysis revealed that the ratio of particles to micrograms of NV proteins was 745.0 ± 51.7 million (Fig. 1c). Also, NVs were generated at a concentration ~ 20 times higher than naturally produced EVs from the same number of cells (Additional file 1: Figure S2). In total, 3536 proteins were identified by mass spectrometry from three biological replicates (Additional file 1: Figure S3), and 12 MSC marker proteins were detected in the NVs (Additional file 1: Table S1). Interestingly, 95 proteins matched the top 100 EV proteins in the EVpedia database for EVs in general [31] (Additional file 1: Table S2). Moreover, the subcellular localization of the identified proteins was examined by GO term analysis to categorize enriched cellular components. The most associated term with the lowest *P* value was “extracellular vesicle” (32.7%), showing that NVs are indeed EV-like structure enriched in EV proteins (Fig. 1d). In addition, we validated that NVs harbor EV marker proteins including Flotillin-1, CD81, and beta-actin, which were more highly enriched in NVs than MSCs lysates (Fig. 1e).

### Functional characterization in NV proteome

**Fig. 2:**
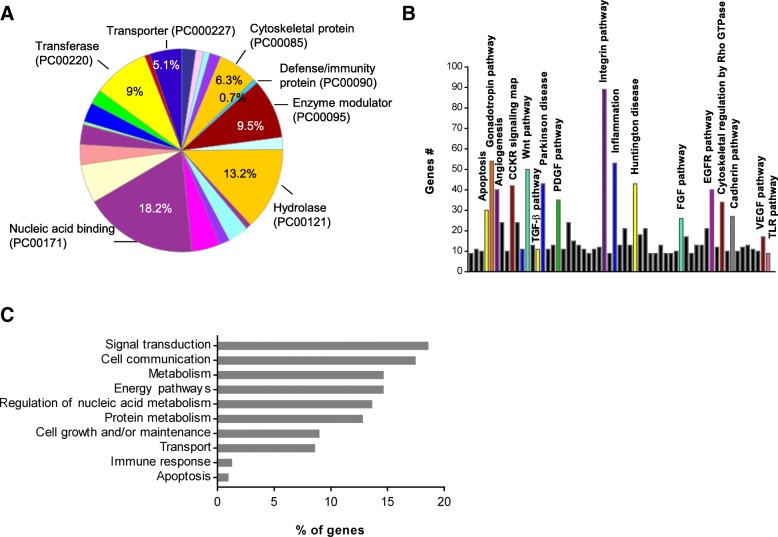
Functional analysis of NV proteome. **a** Distribution of the 2478 proteins sorted from all the NV proteins in different protein class by Panther analysis. **b** Panther pathway analysis of the NV proteome. **c** GO analysis of biological function of NV proteins conducted with Funrich software. The bar chart shows the top ten enriched categories (*P* < 0.05). Three biological replicates were used, and commonly identified proteins in three runs were analyzed for functional analysis

To understand the overall biological function elicited by NV proteins, we performed GO analysis using the Panther tools. Proteins of the NV proteome were primarily incorporated in the following classes: nucleic acid binding, hydrolase, enzyme modulator, transferase, cytoskeletal protein, and transporter (Fig. 2a). Several proteins belonged to defense/immunity protein class although of a low number. Pathway analysis on the NV proteome showed most of the proteins are involved in the following pathways: integrin pathway, inflammation, gonadotropin pathway, and angiogenesis (Fig. 2b). Moreover, the top ten GO biological processes analyzed by the Funrich analysis tool were mainly incorporated in signal transduction, cell communication, metabolism-related process, cell growth, transport, immune response, and apoptosis (Fig. 2c).

### Cellular uptake of NVs in RAW 264.7 cells

**Fig. 3:**
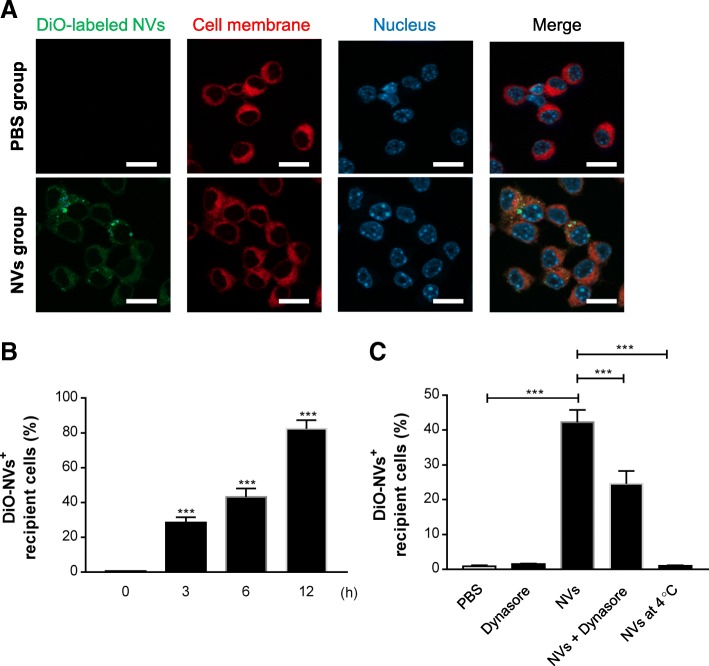
NVs are taken up by macrophage cells via endocytic pathway. **a** NVs (1 × 10^9^) were incubated with RAW 264.7 cells for 6 h. NVs, cell membrane, and nuclei were stained by DiO (green), Cellmask Deep Red (red), and DAPI (blue), respectively. Scale bars, 20 μm. **b** RAW 264.7 cells were treated with DiO-labeled NVs for 0, 3, 6, and 12 h; then the uptake of the labeled NVs by cells was analyzed with flow cytometry; and data show the percentage of DiO-positive cells of three independent experiments. **c** Cells were pretreated with dynasore for 1 h at 37 °C, followed by incubation with DiO-labeled NVs for 6 h at 37 °C. Additionally, NVs were treated to the cells for 6 h at 4 °C. The uptake of the fluorescently labeled NVs by cells was analyzed with flow cytometry, and data represent the percentage of DiO-positive cells of three independent experiments. ***P < 0.001; versus the 0 h group. Error bars indicate SEM

As NVs are expected to exert immune-related function based on the NV proteome analysis, we speculated that immune cells are affected by NVs. Macrophages recognize the presence of invading bacteria as well as secreted vesicles and initiate the host immune response [32]. Thus, we first investigated whether NVs can be taken up by these immune cells to affect immune modulation. The macrophage cell line RAW 264.7 was stained with the membrane-specific stain Cellmask Deep Red before incubation with DiO-labeled NVs. As shown in the confocal micrographs in Fig. 3a, NVs were associated to the cell membranes, but could also be detected inside of cells. We also observed increased NV uptake with prolonged incubation time, shown by FACS analysis (Fig. 3b), which is similar to results by confocal microscopy (Additional file 1: Figure S4). Also, NVs were uptaken by other immune cells such as T cells and B cells over the time; however, the uptake percentage is relatively lower than RAW 264.7 cells (Additional file 1: Figure S5). Moreover, dynasore, an endocytosis inhibitor, significantly blocked entry of the NVs into RAW 264.7 cells (Fig. 3c) and inhibited the transition from M1 into M2 state induced by NVs (Additional file 1: Figure S1C, S1D). Furthermore, there was almost no passive uptake of NVs at 4 °C (Fig. 3c).

### NV-mediated protection against OMV-triggered peritoneal inflammation

**Fig. 4:**
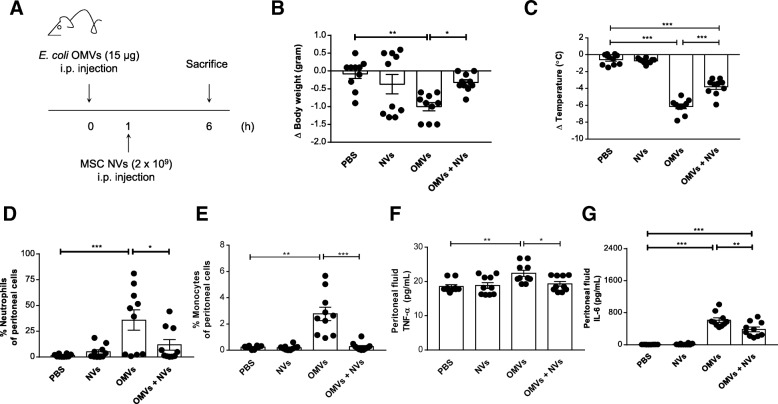
Protective effect of NVs on peritoneal inflammation in OMV-induced sepsis in mice. **a** Study design for investigation of the therapeutic effect of NVs. Sublethal dose of OMVs (15 μg) from *E. coli* was injected i.p. once, followed by i.p. injection of NVs (2 × 10^9^) at 1 h. Six hours after OMV injection, mice were sacrificed to check inflammatory parameters. **b**, **c** Body weight (**b**) and temperature (**c**) were measured at 6 h. **d**, **e** The percentage of neutrophils (**d**) and monocytes (**e**) in the peritoneum were determined by FACS analysis at 6 h following OMV administration. **f**, **g** Peritoneal fluid cytokines such as TNF-α (**f**) and IL-6 (**g**) were measured at 6 h. *n* = 10/group. *P < 0.05; **P < 0.01; ***P < 0.001. Error bars indicate SEM

To address whether MSC-derived NVs may reduce the OMV-mediated cytokine storm in a mouse model, a sublethal dose of *E. coli* OMVs (15 μg) was firstly injected i.p. once to establish the inflammation according to previously reported studies [27, 30]. All mice administered with OMVs showed a significant decrease in body temperature (Additional file 1: Figure S6). Subsequently, NVs (2 × 10^9^) were injected i.p. once at 1 h followed by analysis at 6 h to determine the degree of inflammation, as shown in Fig. 4a. OMV-treated animals showed usually eye exudates, but this phenomenon was reduced by 55% following NV treatment (Additional file 1: Figure S7). Moreover, a decreased pattern in body weight and temperature loss commonly observed after OMV exposure [26] was reduced by NVs at 6 h, albeit not fully reducing hypothermia (Fig. 4b, c).

As intraperitoneal injection of OMVs is associated with acute inflammation in the peritoneum, we quantified inflammatory cells and cytokines in the peritoneal cavity. The number of neutrophils and monocytes, known to mediate inflammation initially following OMV administration [33], was significantly reduced by NV treatment (Fig. 4d, e). Moreover, we found that OMV-induced tumor necrosis factor (TNF)-α and interleukin (IL)-6 increases were meaningfully ameliorated in NV-treated mice (Fig. 4f, g), indicating that post-challenge treatment with NVs can effectively dampen local inflammation activated by OMVs.

### NV-mediated protection against OMV-triggered systemic inflammation

**Fig. 5:**
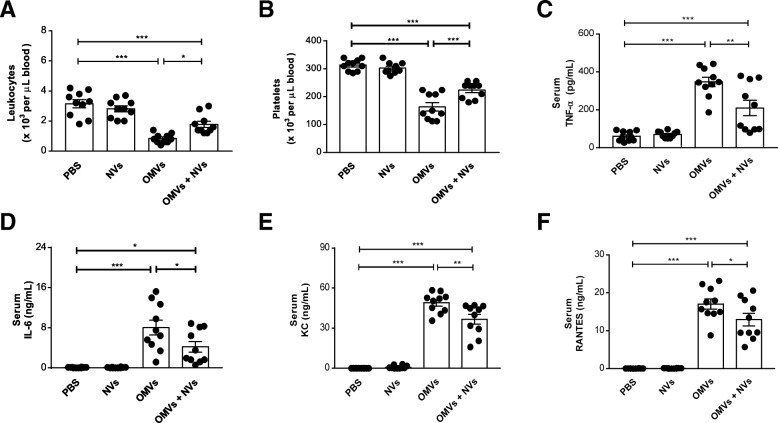
NVs prevent the increase of systemic cytokines and chemokines provoked by OMVs. **a**, **b** The number of leukocytes (**a**) and platelets (**b**) was counted in blood at 6 h following OMV injection. **c**–**f** The effect of NVs on the concentrations of TNF-α (**c**), IL-6 (**d**), KC (**e**), and RANTES (**f**) was analyzed in serum. *n* = 10/group. *P < 0.05; **P < 0.01; ***P < 0.001. Error bars indicate SEM

During the cytokine storm associated with sepsis, systemic inflammatory cells and cytokines are massively increased upon bacterial infection leading to multiorgan failure and death [34]. We therefore next determine any changes in the number of blood leukocytes and platelets representing systemic inflammatory sign. OMV-treated mice showed a substantial decrease in leukocyte and platelet number, whereas it was greatly improved by NV treatment (Fig. 5a, b). We further explored whether MSC-derived NVs could show therapeutic effects with regard to systemic cytokines and chemokines. The cytokine antibody array in the serum showed a remarkable change in cytokines and chemokine expression pattern between the group only treated with OMVs and the group treated also with NVs (Additional file 1: Figure S8). Especially, the administration of NVs significantly reduced the elevation of TNF-α and IL-6 levels seen after OMV exposure (Fig. 5c, d). Moreover, OMV-induced KC and RANTES, chemoattractants for neutrophils and macrophages, respectively, were also diminished by the treatment of NVs (Fig. 5e, f).

Given that OMVs can exert a strong inflammatory effect on a distant organ such as the lung [26], we next determine whether the NVs had any anti-inflammatory effects in this organ, by quantifying inflammatory cells and cytokines in BAL fluid. Similar to results obtained in the blood, MSC-derived NVs protected against OMV-induced increase in the level of infiltrated cells (Additional file 1: Figure S9A), TNF-α (Additional file 1: Figure S9B), and IL-6 (Additional file 1: Figure S9C), suggesting that NVs may have a therapeutic role in the treatment of systemic inflammation as well as distant organ inflammatory responses.

### In vivo distribution of NVs in mice

**Fig. 6:**
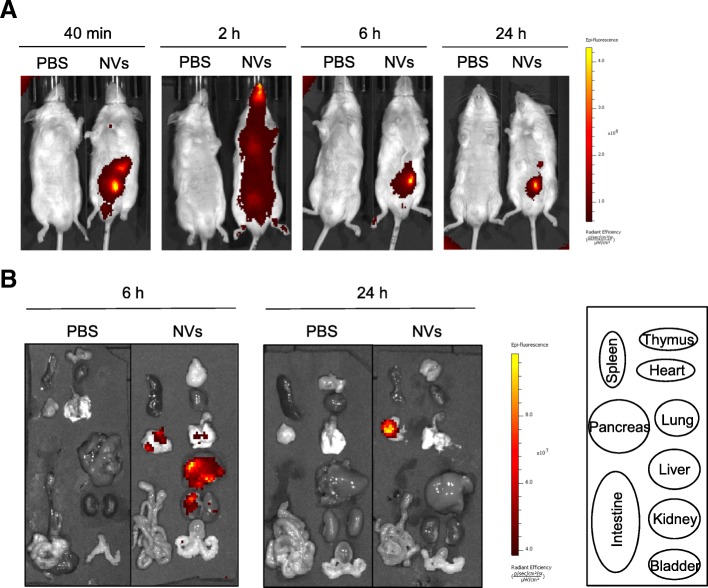
Biodistribution kinetic analysis of NVs in mice with near-infrared imaging. **a** The mice were injected by intraperitoneal injection of Cy7-labeled NVs (2 × 10^9^) or PBS. At each time point, the fluorescence of the whole body was obtained by IVIS spectrum. **b** Various tissues were obtained at 6 and 24 h following injection of Cy7-labeled NVs. *n* = 3/group

To explore the mechanism by which MSC-derived NVs exert an influence on long-distance systemic inflammation, we analyzed the biodistribution of NVs in mice by accomplishing near-infrared (NIR) imaging, known to be capable of deep tissue visualization characterized by minimally invasive and sensitive technique [35]. The whole body distribution of NVs was analyzed after i.p. injection of the same dose of Cy7-labeled NVs as used in the precedent inflammatory experiments (Figs. 4 and 5). NIR imaging revealed that NV signals were only detected in the peritoneal area at the early time, and then widely extended to the whole body with the highest signals at 2 h (Fig. 6a). After 2 h and up to 24 h, the signals gradually decreased but still remained in the peritoneum area at 24 h after injection. Mice were sacrificed to further examine Cy7 signals in various tissues at 6 h and 24 h after NV injection. Strong Cy7 signals were observed in the liver, kidney, pancreas, and especially lung at 6 h (Additional file 1: Figure S10), and then disappeared at 24 h (Fig. 6b), implying that NVs may have the possibility to give a protective effect against systemic inflammation in various organs.

### Blockade of IL-10 action negates the inhibitory effects of NVs

**Fig. 7:**
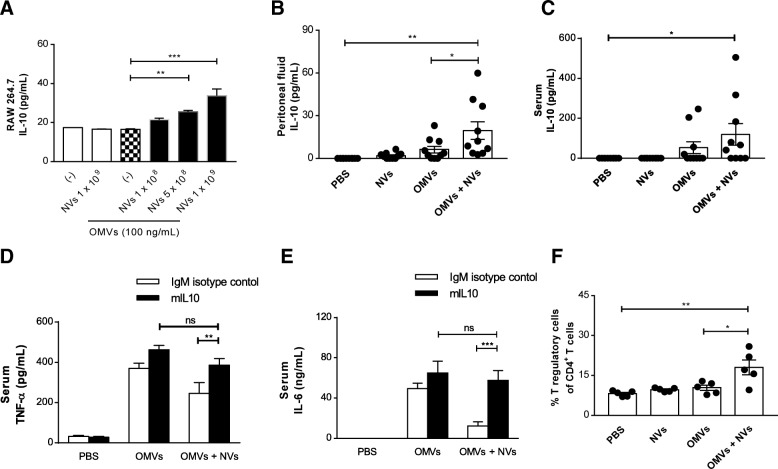
IL-10-mediated immune suppression of NVs in OMV-induced sepsis. **a** RAW 264.7 cells were pre-incubated with OMVs (100 ng/mL) for 3 h and treated with various doses of NVs for 15 h, and the concentration of IL-10 in the conditioned media was measured. *n* = 3/group. **b**, **c** The concentration of IL-10 was evaluated in the peritoneal fluid (**b**) and serum (**c**) at 6 h following OMVs administration. *n* = 10/group. **d**, **e** mIL-10 or isotype-matched control antibody was intraperitoneally injected together with OMVs, followed by injection NVs after 1 h. Serum concentrations of TNF-α (**d**) and IL-6 (**e**) were measured at 6 h. *n* = 5/group. **f** The percentage of T regulatory cells in the peritoneum were determined by FACS analysis at 6 h. *n* = 5/group. *P < 0.05; **P < 0.01; ***P < 0.001; ns, not significant. Error bars indicate SEM

To identify the mechanism by which MSC-derived NVs suppress OMV-induced inflammation, we firstly used OMV-stimulated macrophages as a cell model of inflammation. Pro-inflammatory cytokines TNF-α and IL-6 were markedly increased in OMV-treated mouse macrophages compared to the control group (Additional file 1: Figure S1E, S1F). However, NV treatment dose-dependently reduced OMV-induced cytokine secretion, which is consistent with our in vivo findings in the mouse model as mentioned above. Also, NVs significantly reduced the secretion of pro-inflammatory cytokines from primary mouse macrophages and human macrophages activated by the OMVs (Additional file 1: Figure S11, S12). Interestingly, IL-10 levels were dose-dependently upregulated in OMV-stimulated macrophages after NV treatment, whereas OMVs alone did not change IL-10 production (Fig. 7a). Also, NVs could more increase IL-10 secretion from macrophages pre-activated with OMVs for a longer time (Additional file 1: Figure S13).

Given that IL-10 has been recognized as an effective anti-inflammatory reagent for bacterial infection [36, 37], we determined whether IL-10 was present in the peritoneal fluid and serum collected from NV-treated mice at 6 h post-OMV administration. Mice treated with only NVs or OMVs did not have any changes in the levels of IL-10 in peritoneal fluid (Fig. 7b) and serum (Fig. 7c). However, treatment of OMV-injected mice with NVs significantly upregulated the IL-10 levels in both peritoneal fluid and serum. To further confirm the role of IL-10 in the NV-induced therapeutic in vivo model, mice were injected with an anti-IL-10 monoclonal antibody (mIL-10) or isotype-matched control antibody. NV-mediated protection of mice from OMV-induced cytokine production could be dampened by prior administration of mIL-10 in the serum (Fig. 7d, e, filled bars). However, this attenuating effect was not observed by the control antibody, verifying the specificity of this effect (Fig. 7d, e, open bars). IL-10 has been known to be an important mediator for immunosuppressive cells such as T regulatory cells and myeloid-derived suppressive cells [38]. Interestingly, the infiltration of T regulatory cells into the peritoneum was significantly increased by NV treatment (Fig. 7f) although there was no change in the proportion of myeloid-derived suppressive cells (Additional file 1: Figure S14). These results indicate that endogenously expressed IL-10 is involved in the NV-induced immunomodulatory effects.

## Discussion

In this study, we show that NVs derived from MSCs are enriched in proteins released by natural EVs and have protective immunomodulatory effects in vitro as well as in a mouse model of sepsis provoked by bacterial OMVs. Specifically, we demonstrate that the NVs can be taken up by macrophages and significantly reduce the production of pro-inflammatory cytokines from the cells activated by the OMVs*.* These findings are further validated in vivo, where we show that NVs ameliorate the signs of cytokine storm, including body weight and temperature changes, as well as the excessive inflammatory response. In addition, intraperitoneally injected NVs distribute to the whole mouse body and accumulate in the lung, liver, and kidney within a short period of time, where they also exert an effect. Moreover, IL-10 is an important anti-inflammatory mediator in NV-induced protectivity in mice. These results in combination support the hypothesis that MSC-derived NVs can contribute to reduce the OMV-induced systemic inflammation in vitro and in vivo via IL-10 release.

When NVs are generated from bone marrow-derived MSCs, they show similar morphology and diameter as natural EVs, as compared to previous studies [18, 19] (Fig. 1a, b). We here show, by mass spectrometric analysis and Western blot, that MSC-derived NVs carry a vast number of proteins that are present in naturally released EVs, including Flotillin-1 and CD81 (Figs. 1 and 2). Importantly, our proteomic analysis shows that the NVs also carry several prototypical MSC markers, which have also been identified in an earlier MSC-EV proteomic study [39], as well as 95 proteins of the top 100 EV markers in the EVpedia database [31]. This suggests that NVs originate from parental MSCs and closely resemble EVs in vesicular composition. We also identified that NV proteins were primarily incorporated as nucleic acid binding, hydrolase, enzyme modulator, transferase, cytoskeletal protein, and transporter, recently linked to MSC-derived EV proteins [39, 40]. Host defense/immunity proteins were also detected in the NV proteome, implying relation with immunomodulation. For example, NVs included syndecan 4, CD109, alpha-2-macroglobulin, and programmed cell death 1 ligand 1, which are known to be associated with immunosuppression and regulation of repair process [41–44]. Moreover, based on pathway analysis and GO biological enrichment analysis, NV proteins appear to connect closely to the inflammatory cytokine pathway.

MSC-derived EVs have shown to participate in many physiological and pathological processes mediated by vesicular membrane proteins, cytosolic proteins, mRNAs, and miRNAs [45, 46]. Especially, it is increasingly recognized that EV-associated proteins are important effectors for MSC-derived EV-mediated beneficial activities. Jagged-1 ligand protein from EVs was involved in EV-mediated angiogenesis for ischemic recovery [47]. Also, CD73 present on EVs was shown to repair osteochondral defects in chondrocytes and facilitate induction of an anti-inflammatory phenotype of macrophages [48]. Further, delivery of 14-3-3 protein via EVs prevented tubule epithelial cells injury induced by the chemotherapy drug cisplatin [49]. Interestingly, these referred proteins were also observed in NV proteome (data not shown), suggesting that the contribution of a certain NV protein to induce an anti-inflammatory response may be necessarily elucidated in further studies.

EVs need to interact with target cells through fusion with cell plasma membrane to deliver their cargo molecules inside the recipient cell cytoplasm. Cells are known to take up EVs via multiple mechanisms, including endocytosis such as clathrin-mediated endocytosis, macropinocytosis, phagocytosis, and lipid raft-mediated internalization [50]. Recently, Shimoda et al. reported that the uptake of EVs from human adipose-derived MSCs was blocked with antibody against siglec (CD33) on recipient cells, confirming EV uptake via sialic acid-siglec interaction [51]. It is also found that the interaction of Thy-1 (CD90) on MSC-derived EVs with beta integrins mediates internalization of EVs into lung fibroblasts [52]. Interestingly, Thy-1 was also found in NVs, implying that this protein could contribute to NV internalization (Additional file 1: Table S1). However, the exact mechanism by which MSC-derived NV are taken up has not been characterized. We here confirmed the endocytic internalization of MSC-derived NVs into macrophages using dynasore, an inhibitor for dynamin (Fig. 3c). Dynamin is a GTPase required for the clathrin-mediated endocytosis, and inhibition of the protein interrupted almost all EV internalization activity in a variety of cells [53, 54]. Similarly, dynasore significantly prohibited entry of the NVs into macrophages, suggesting the involvement of clathrin-mediated uptake, although the effect with dynasore is not complete. Kim et al. have shown that adipose stem cell-derived NVs are internalized into airway epithelial cells by various pathways, especially receptor-mediated endocytosis depending on active heparan sulfate proteoglycan on recipient cells [21]. Thus, the relative importance of other internalization pathways for NVs needs to be elucidated.

As MSC-derived EVs contain immunomodulatory activities mediated by vesicular proteins and genetic materials, EV-based therapeutics have been well documented in many animal disease models [45]. Although the protective effects of EVs have been demonstrated in severe inflammatory conditions [14, 15], there are still challenges for clinical use due to the limitation in low yield and complex purification processes [16, 17]. Ultracentrifugation and precipitation have been used for greater yield, but the methods might affect the integrity and activity of EVs with a high risk of association with protein aggregates [55, 56]. In order to overcome these issues, NV generation through serial extrusion through filters has been recently developed for the development of therapeutics with high potency and productivity in cancer, diabetes, and emphysema models [18, 20, 21]. For example, MSC-derived NVs have been found to be generated with a 30 times higher yield than natural EVs and have regenerative effects comparable to EVs in emphysema [21]. In the same context, we observed a 20 times higher yield of NVs versus EVs, and the produced NVs efficiently prevented the excessive inflammation and cytokine storm in OMV-induced septic mice (Figs. 4 and 5), similarly to what has been observed with MSC-derived EVs in sepsis models [14, 15]. In this specific series of experiments, we did not directly compare the effects of NVs and EVs with regard to in vivo activities; however, it is expected that both NVs and EVs induce an anti-inflammatory response in OMV-induced sepsis in a similar fashion because they share many bioactive vesicular molecules.

Our study showed that intraperitoneally injected NVs diminish the systemic inflammatory response in the blood and lung provoked by OMV administration (Fig. 5). The systemic effects may be mediated by the MSC NV biodistribution throughout the body, or alternatively via secondary effects on activated host cells upon NV uptake. Jang et al. have previously reported that macrophage-derived NVs can distribute throughout the body, but especially accumulated in tumor tissue, confirming tissue-specific localization [18]. Consistent with previous observations, NIR imaging of NV signals showed that intraperitoneally injected NVs were spread to the whole body within early time and localized in various organs such as the lung, liver, and kidney (Fig. 6). This suggests that when NVs are administrated in the peritoneum, they may easily move across the intestinal barrier to reach other tissues to induce anti-inflammatory effects elsewhere. However, it remains to be established whether NVs are delivered to the blood directly or via immune cells taking up NVs.

In addition to plausible direct NV effect, secondary mediators from immune cells activated by NVs may contribute to NV-mediated protectivity against sepsis. Berg et al. previously reported that IL-10 knockout mice increase susceptibility to lethal dose of endotoxic shock, arguing that IL-10 is a critical component of the host’s natural defense in a situation of cytokine storm [57]. Also, administration of IL-10 after induction of sepsis was found to decrease lethality in mice through suppressing the elevation of systemic TNF-α [37]. NVs induced IL-10 production in OMV-activated macrophages (Fig. 7a), which is consistent with the previous study showing that macrophage-derived IL-10 is necessary for the protective effect of MSCs in sepsis [3]. In addition, in the peritoneal fluid and serum of OMV-induced sepsis mice, NV treatment increased IL-10 secretion both locally and systemically (Fig. 7b, c). Likewise, MSC-derived EVs were also shown to upregulate IL-10 levels after LPS stimulation [58], implying that NVs may have a similar protective mechanism to EVs. We next assessed the effect of mIL-10 administration on NV-mediated protection in mice. NV-downregulated pro-inflammatory cytokines such as TNF-α and IL-6 in serum were abolished by mIL-10 in OMV-treated mice (Fig. 7d, e), suggesting that IL-10 may be considered crucial secondary immunomodulator promoted by NVs.

To date, most sepsis studies on investigative therapeutic molecules have been exploited in a mouse model established with LPS or bacteria inoculation. We previously published data showing that OMV-induced mortality and cardiac dysfunction was significantly higher than LPS alone [26, 27], indicating that OMVs cause inflammation also via other mechanisms than LPS. Although bacteria themselves are generally considered as an essential cause of infection, in fact, severe septic patients only rarely turn out to have positive blood cultures [59]. Also, OMVs have been detected in the blood from a septic patient [60] and can themselves mimic sepsis [61].

## Conclusions

Our study demonstrates, for the first time, the protective effect of NVs on OMV-activated macrophages and septic mice and describes the role of IL-10 in the NV-mediated anti-inflammatory response. Further, our collective data may argue that EV-mimetic NVs from MSCs may have economic advantages due to high yield and may be efficiently therapeutic vehicles incorporating other anti-inflammatory agents. Future development of NVs derived from various cell and tissue sources should be considered to be clinically applicable to many human diseases including sepsis.

## Additional file

Additional file 1: **Figure S1.** NV treatment dose-dependently inhibits OMVs-induced cytokines in RAW 264.7 cells. **Figure S2.** NV production yield is much higher than EVs from MSCs. **Figure S3.** A Venn diagram shows the overlap between NV proteins identified in three biological replicates. **Figure S4.** NVs are taken up by macrophage cells time-dependently. **Figure S5.** NVs are taken up by adaptive immune cells. **Figure S6.** Hypothermia is induced by injection of OMVs in mice. **Figure S7.** A decrease in the formation of eye exudates under the treatment of NVs. **Figure S8.** Overall NV-induced cytokines and chemokine expression profile in serum of OMV-treated mice. **Figure S9.** NVs prevent the increase of BAL cells and cytokines induced by OMVs. **Figure S10.** Biodistribution analysis of NVs in mice with near-infrared imaging. **Figure S11.** NV treatment reduces OMV-induced pro-inflammatory cytokines in mouse peritoneal macrophages. **Figure S12.** NV treatment decreases OMV-induced cytokines in human macrophages. **Figure S13**. NV treatment increases OMV-induced IL-10 in macrophages. **Figure S14.** NV treatment does not affect myeloid-derived suppressor cells (MDSCs) infiltration. **Table S1.** MSC markers expressed in NVs. **Table S2.** EV markers expressed in NVs. (DOCX 11210 kb)

## Data Availability

The datasets used and/or analyzed during the current study are available from the corresponding author on reasonable request.

## Abbreviations

BAL: Bronchoalveolar lavage
GO: Gene ontology
IL: Interleukin
LPS: Lipopolysaccharides
MSCs: Mesenchymal stromal cells
NVs: Nanovesicles
OMVs: Outer membrane vesicles
TEM: Transmission electron microscopy
TNF: Tumor necrosis factor
