# Supplementary material for: Mesenchymal stromal cell-derived nanovesicles ameliorate bacterial outer membrane vesicle-induced sepsis via IL-10
Source: Stem Cell Res Ther. 2019 Aug 1;10:231. doi: 10.1186/s13287-019-1352-4 (PMC6676541; doi:10.1186/s13287-019-1352-4)
Supplement: Supplementary file 1 — Figure S1. NV treatment dose-dependently inhibits OMVs-induced cytokines in RAW 264.7 cells. Figure S2. NV production yield is much higher than EVs from MSCs. Figure S3. A Venn diagram shows the overlap between NV proteins identified in three biological replicates. Figure S4. NVs are taken up by macrophage cells time-dependently. Figure S5. NVs are taken up by adaptive immune cells. Figure S6. Hypothermia is induced by injection of OMVs in mice. Figure S7. A decrease in the formation of eye exudates under the treatment of NVs. Figure S8. Overall NV-induced cytokines and chemokine expression profile in serum of OMV-treated mice. Figure S9. NVs prevent the increase of BAL cells and cytokines induced by OMVs. Figure S10. Biodistribution analysis of NVs in mice with near-infrared imaging. Figure S11. NV treatment reduces OMV-induced pro-inflammatory cytokines in mouse peritoneal macrophages. Figure S12. NV treatment decreases OMV-induced cytokines in human macrophages. Figure S13. NV treatment increases OMV-induced IL-10 in macrophages. Figure S14. NV treatment does not affect myeloid-derived suppressor cells (MDSCs) infiltration. Table S1. MSC markers expressed in NVs. Table S2. EV markers expressed in NVs. (DOCX 11210 kb) [file 13287_2019_1352_MOESM1_ESM.docx]

**Additional file 1**

**Figure S1. NVs treatment dose-dependently inhibit OMVs-induced cytokines in RAW 264.7 cells. (A and B)** RAW 264.7 cells were pre-incubated with OMVs for 3 h, and treated with three batches of NVs (1 × 10^9^) for 15 h, and the concentration of cytokines in the conditioned media was measured. **(C and D)** RAW 264.7 cells were pre-incubated with OMVs for 3 h, and treated with NVs (1 × 10^9^) together with Dynasore for 15 h, and the concentration of TNF-α **(C)**, and IL-10 **(D)** in the conditioned media was measured. *n* = 3 / group. **(E and F)** RAW 264.7 cells were pre-incubated with OMVs for 3 h, and treated with NVs for 15 h, and the concentration of cytokines in the conditioned media was measured. *, *P*<0.05; **, *P*<0.01; ****, P*<0.001; ns, not significant; versus (-) group. Error bars indicate SEM.

**Figure S2. NV production yield is much higher than EVs from MSCs.** The number of particles was measured by nanoparticle tracking analysis from EVs and NVs. *n* = 3 / group. ****, P*<0.001. Error bars indicate SEM.

**Figure S3. A Venn diagram shows the overlap between NV proteins identified in three biological replicates.** A total of 3,536 proteins were overlapped from three replicates.

**Figure S4. NVs are taken up by macrophage cells time-dependently.** NVs (1 × 10^9^) were incubated with RAW 264.7 cells for 0 (control), 3, 6, and 12 h. NVs, cell membrane, and nuclei were stained by DiO (green), Cellmask Deep Red (red), and DAPI (blue), respectively. Scale bars, 20 µm.

**Figure S5. NVs are taken up by adaptive immune cells.** **(A and B)** T cells **(A)** and B cells **(B)** were treated with DiO-labelled NVs (1 × 10^9^) for 0, 3, 6, and 12 h, and then the uptake of the labelled NVs by cells was analyzed with flow cytometry, and data show the percentage of DiO-positive cells of three independent experiments. **, *P*<0.1; ****, P*<0.001; versus 0 h group. Error bars indicate SEM.

**Figure S6. Hypothermia is induced by injection of OMVs in mice.** Sublethal dose of OMVs (15 µg) from *E. coli* was injected i.p. once, followed by measurement of body temperature at 1 h. *n* = 10 / group. ****, P*<0.001; ns, not significant. Error bars indicate SEM.

**Figure S7. A decrease in the formation of eye exudates under the treatment of NVs. (A and B)** OMV-induced eye exudates were recovered by NVs at 6 h following OMV injection **(A)** and the percentage value of mice developing eye exudates from 10 mice was shown **(B)**.

**Figure S8. Overall NV-induced cytokines and chemokines expression profile in serum of OMV-treated mice.** The expression pattern of total 40 cytokines and chemokines was investigated by cytokine antibody array in serum at 6 h following OMV injection. Color code indicates mean pixel density of the expressed proteins (red, most expressed; green, least expressed)

**Figure S9. NVs prevent the increase of BAL cells and cytokines induced by OMVs.** **(A-C)** The number of total cells **(A)**, TNF-α **(B)**, and IL-6 **(C)** were examined in BAL fluid at 6 h following OMV injection. *n* = 10 / group. *, *P*<0.05; **, *P*<0.01; ****, P*<0.001. Error bars indicate SEM.

**Figure S10. Biodistribution analysis of NVs in mice with near-infrared imaging.** Radiant efficiency was measured using Living image 3.1 software at 6 h. n = 3 / group. **, *P*<0.01; ****, P*<0.001; versus PBS group. Error bars indicate SEM.

**Figure S11. NV treatment reduces OMV-induced pro-inflammatory cytokines in mouse peritoneal macrophages. (A-C)** Macrophages were pre-incubated with OMVs (100 ng/mL) for 3 h, and treated with NVs (1 × 10^9^) for 15 h, and the concentration of TNF-α **(A)**, IL-6 **(B),** and IL-10 **(C)** in the conditioned media was measured. *n* = 3 / group. *, *P*<0.05; ****, P*<0.001. Error bars indicate SEM.

**Figure S12. NV treatment decreases OMV-induced cytokines in human macrophages. (A and B)** Differentiated U937 cells were pre-incubated with OMVs (100 ng/mL) for 3 h, and treated with NVs (1 × 10^9^) for 15 h, and the concentration of TNF-α **(A)** and IL-6 **(B)** in the conditioned media was measured. *n* = 3 / group. ****, P*<0.001. Error bars indicate SEM.

**Figure S13. NV treatment increases OMV-induced IL-10 in macrophages.** RAW 264.7 cells were pre-incubated with OMVs (100 ng/mL) for 24 h, and treated with NVs (1 × 10^9^) for 15 h, and the concentration of IL-10 in the conditioned media was measured. *n* = 3 / group. ****, P*<0.001. Error bars indicate SEM.

**Figure S14. NV treatment does not affect myeloid-derived suppressor cells (MDSCs) infiltration.** Sublethal dose of OMVs (15 µg) from *E. coli* was injected i.p. once, followed by i.p. injection of NVs (2 × 10^9^) at 1 h. Six hours after OMV injection, the percentage of MDSCs in peritoneum was determined by FACS. *n* = 5 / group. ns, not significant. Error bars indicate SEM.

**SUPPLEMENTARY FIGURES**

**Figure S1**

**
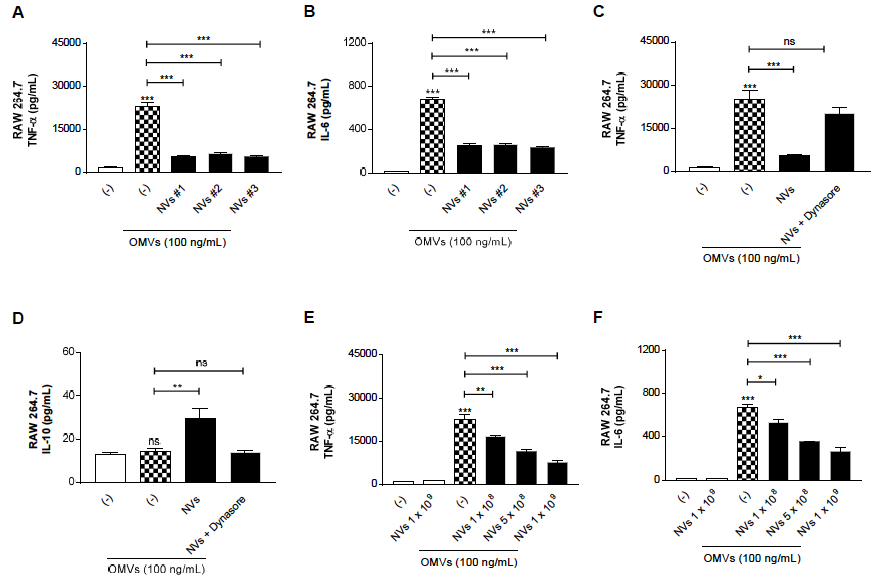
**

**Figure S2**

**
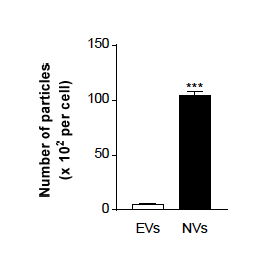
**

**Figure S3**


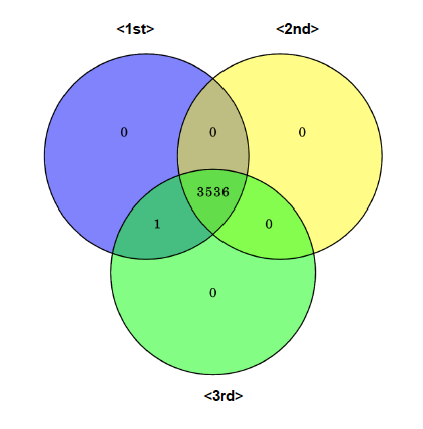


**Figure S4**

**
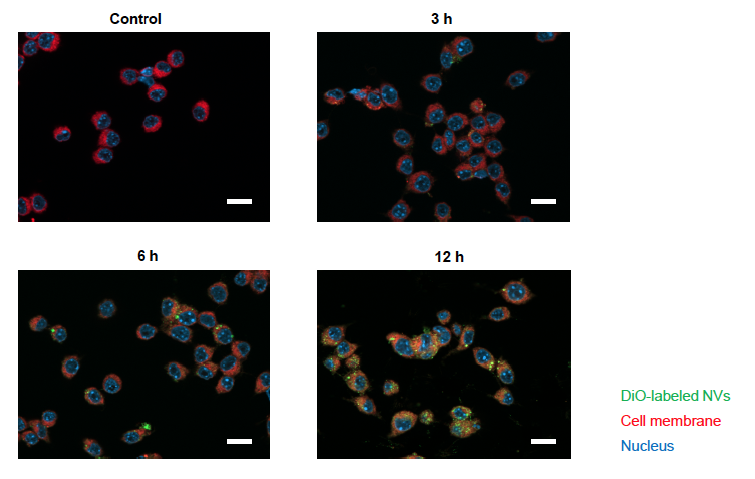
**

**Figure S5**

**
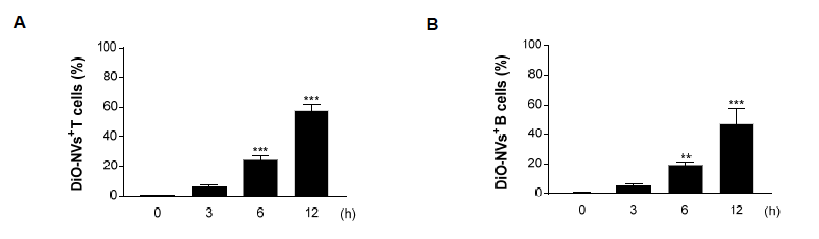
**

**Figure S6**

**
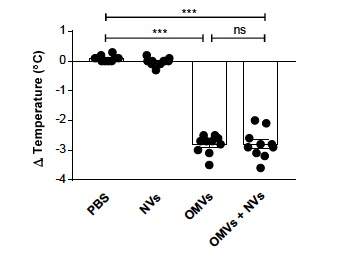
**

**Figure S7**


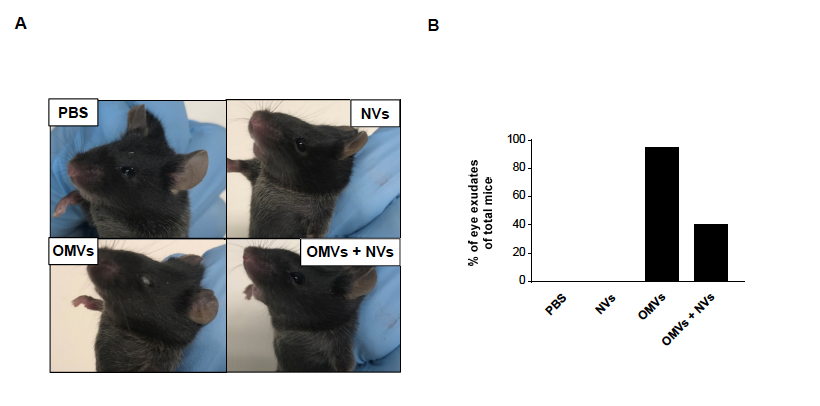


**Figure S8**


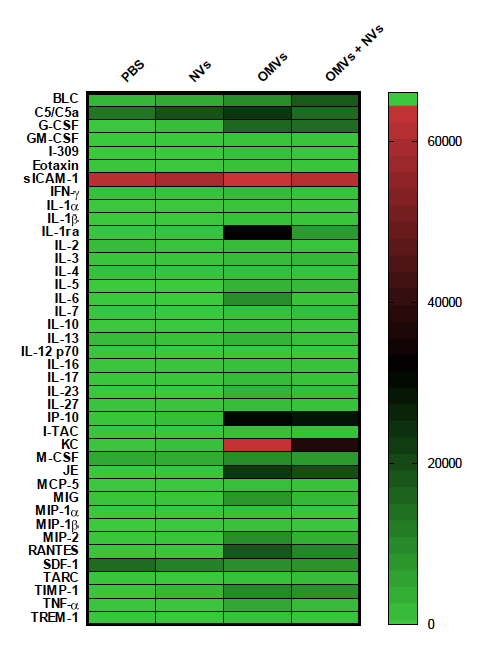


**Figure S9**


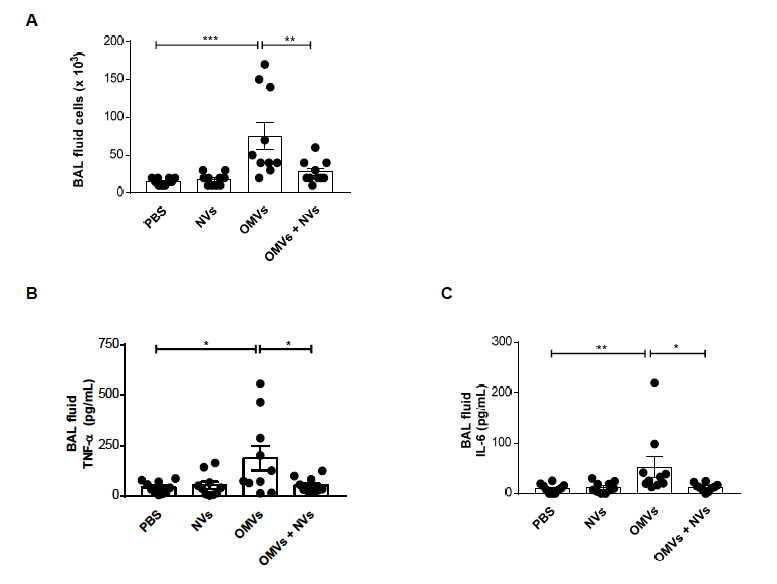


**Figure S10**


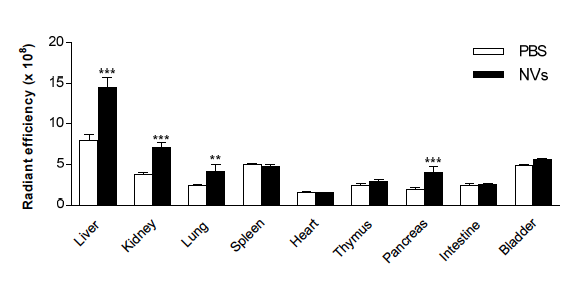


**Figure S11**


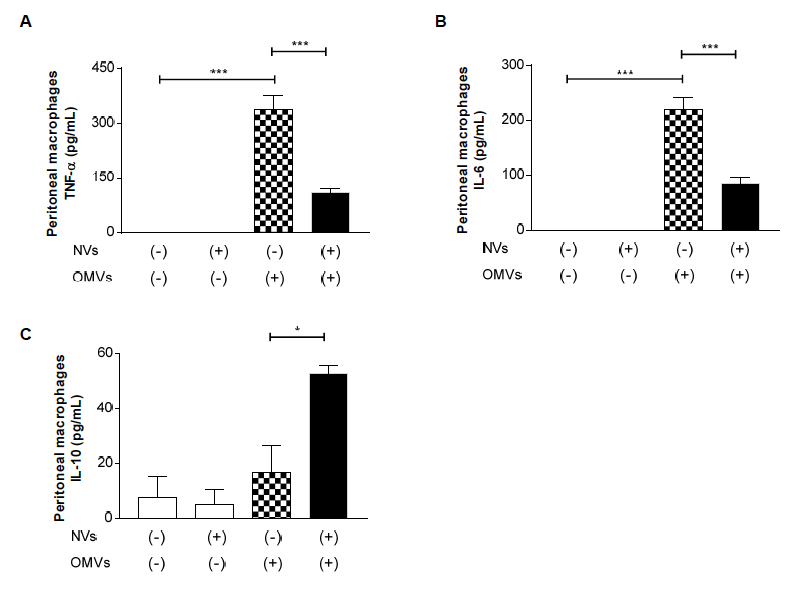


**Figure S12**


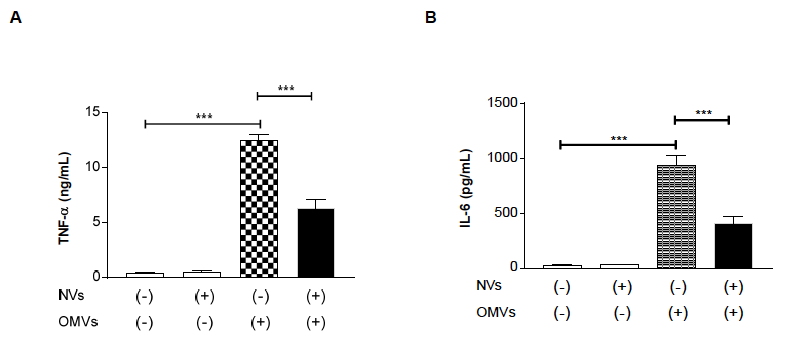


**Figure S13**


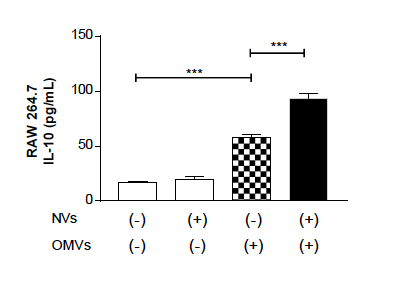


**Figure S14**


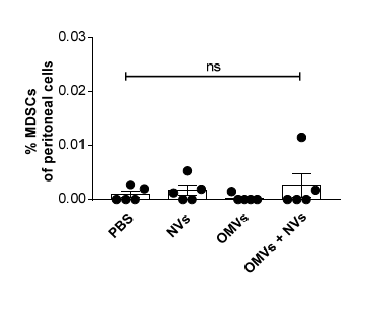


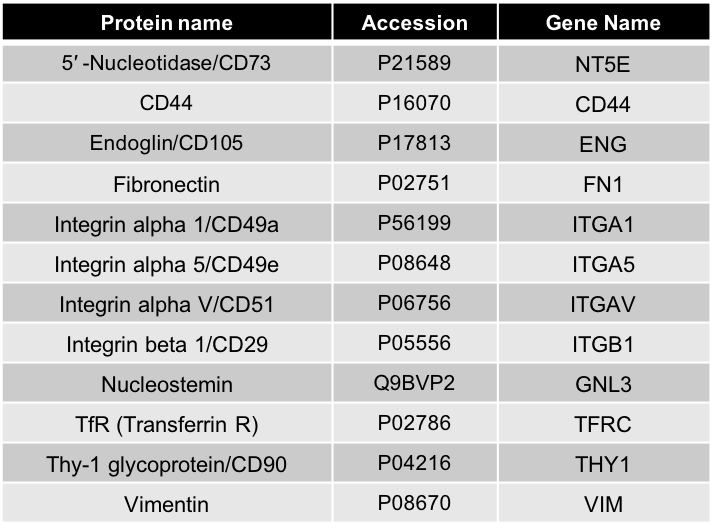


**Table S1. MSC markers expressed in NVs.**

**
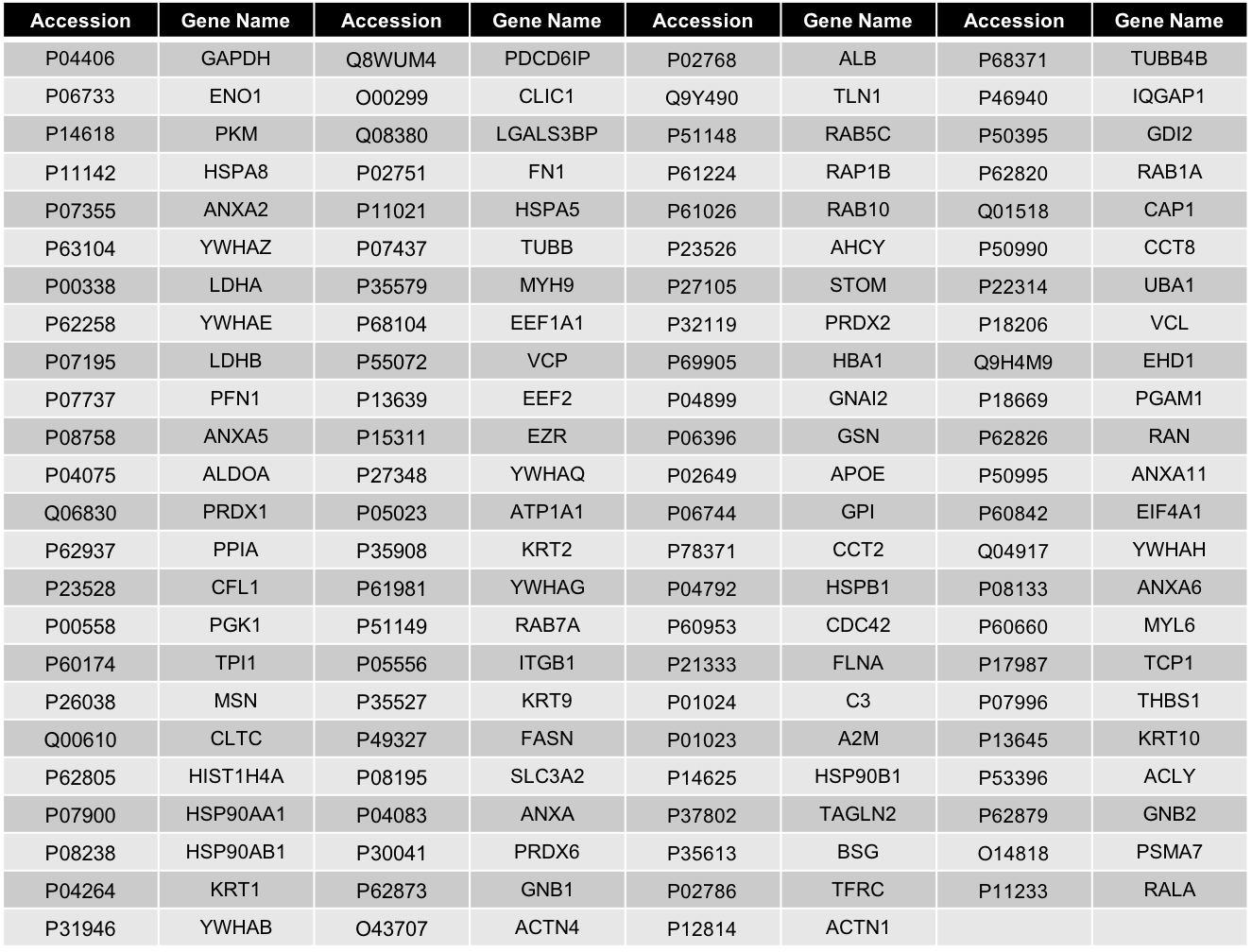
**

**Table S2. EV markers expressed in NVs.**
